# New Species of *Grotea* (Hymenoptera, Ichneumonidae, Labeninae) from Ecuador, with New Records and a Key to the Neotropical Species

**DOI:** 10.1007/s13744-024-01166-1

**Published:** 2024-07-09

**Authors:** Marina Mazón, Santiago Bordera, Gavin R. Broad

**Affiliations:** 1 Departamento de Ciencias Ambientales y Recursos Naturales, Universidad de Alicante, Apdo. Corr. 99, 03080 Alicante, Spain; 2 Centro de Investigaciones Tropicales del Ambiente y Biodiversidad – CITIAB, Universidad Nacional de Loja, Loja, Ecuador; 3 The Natural History Museum, Cromwell Road, London, SW7 5BD UK

**Keywords:** Parasitoid wasp, Darwin wasp, South America, taxonomy

## Abstract

Here we describe two new *Grotea* species from Ecuador, *G. akakana* Mazón & Bordera **sp. nov.,** and *G. romeri* Mazón **sp. nov.**, as well as the male of *G. cundinamarquesa* Herrera-Flórez 2018. *G. akakana* **sp. nov.** is characterized by the combination of a postgenal process long, a 45-flagellomeres antenna without a white band and a mesopleuron black with two yellow spots separated by a red one. On the other hand, *G. romeri* **sp. nov.** is characterized by the combination of a postgenal process very short, a 36-flagellomeres antenna without a white band, a propodeum with a long and narrow area lateralis, uninterrupted yellow-colored orbits and a mesopleuron black with a yellow spot in the middle. The species *G. santandereana* Herrera-Flórez 2018 and *G. surinamese* Herrera-Flórez 2019 are recorded from Ecuador for the first time. This brings the total of described *Grotea* species to 31, all from the New World, with 27 of these exclusively Neotropical. A key for the identification of Neotropical species is included.

## Introduction

*Grotea* is a distinctive genus of Labeninae, defined mainly by the medial projection of the hypostomal carina (i.e., postgenal process); the insertion of the hind coxae in the same plane as the metasoma; and by the long first metasomal tergite with the spiracle close to the middle (Gauld and Wahl 1999). Where the biology is known, *Grotea* species are cleptoparasitoids of bees nesting in hollow twigs, with unusual larval habits for the family Ichneumonidae, as successive larvae of the host bees are killed and eaten, along with the pollen mass (e.g., Graenicher 1905; Gauld 2000).

The genus is widely distributed across the New World: 29 species of *Grotea* have been described so far, mostly from the Neotropics, with only four species occurring in the Nearctic region: *G. anguina* Cresson, 1864, *G. californica* Cresson, 1879, *G. lokii* Slobodchikoff 1970 and *G. mexicana* Cresson, 1874 (Yu et al. 2016; Herrera-Flórez 2018; Herrera-Flórez and Penteado-Dias 2019; Sandoval and Santos 2021; Lima and Kumagai 2024). Although eleven Neotropical species have been described in the last ten years (Herrera-Flórez 2014, 2018; Herrera-Flórez and Penteado-Dias 2019; Sandoval and Santos 2021; Lima and Kumagai 2024), no revision or complete review of the genus has been published since Slobodchikoff (1970).

In Ecuador, only one *Grotea* species has been recorded previously, *G. fulva* Cameron, collected in 1965 in Orellana province (Northern Ecuadorian Amazon) (Slobodchikoff 1970). Here we provide details of two additional species found in Ecuador and describe two new species, as well as the first description of the male of *G. cundinamarquesa* Herrera-Flórez. Furthermore, we include a key to all Neotropical species of *Grotea*.

## Material and methods

Most specimens here studied were collected by the first author during six years of sampling in the southern region of Ecuador (Loja, El Oro and Zamora Chinchipe provinces), comprising three types of ecosystems: tropical dry forest, Andean forest and rainforest. Collecting sites were all in protected areas (either private or government), including three types of habitats with different conservation status: well-conserved forests, areas being restored over the past 10-15 years, and degraded areas (pasture-like areas). All these specimens are deposited in LOUNAZ, the collection of Universidad Nacional de Loja (Ecuador). For more details about these collection areas, see Mazón et al. (2023).

Furthermore, we revised all Labeninae material from Pontificia Universidad Católica de Quito collection (QCAZ), from Quito, as well as from the Natural History Museum (NHMUK) in London. Keys were made based on the keys and descriptions from Slobodchikoff (1970), Porter (1989), Herrera-Flórez (2014, 2018), Herrera-Flórez and Penteado-Dias (2019), Sandoval and Santos (2021) and Lima and Kumagai (2024), as well as from material deposited in NHMUK. We include high quality images of the holotype of *Grotea cortesi* Porter 1989, from the American Entomological Institute Collection (Utah State University, Logan, USA), since this species has not been included in previous identification keys and the original description was rather brief. Also, a key to all Neotropical species described to date is included. Although traditional biogeographic regions include southern South America in the Neotropical Region, various taxonomists have recognised the distinctness of this temperate fauna and flora, and Porter (1998) used the term ‘Neantarctic’. However, in keeping with the regions used by zoologists, we are considering all Central and South America and the tropical region of Mexico as the Neotropics.

For consistency with the most complete prior works on *Grotea*, morphological terminology follows that of Slobodchikoff (1970) and Gauld (2000). Terminology used for describing sculpture is based on Eady (1968). Layer (extended focus) photographs were taken at NHMUK using a Canon EOS 5DSR digital camera with a Canon MP-E 65 mm Macro Lens attached to a StackShot Macro Rail system controlled by Helicon Remote and Helicon Focus ver. 3.6.6W. Images from Utah State University were taken using an EntoVision micro-imaging system.

Species keys are for females, but most characters are compatible for males.

New distribution records of each species are marked with an asterisk.

## Results

### Treatment of species

#### New species

##### *Grotea akakana* Mazón & Bordera, sp. nov.

**Diagnosis:** *Grotea akakana* **sp. nov.** can be distinguished from all described species of the genus by the following combination of characters: postgenal processes long, almost touching; antenna with about 45 flagellomeres, without a white band; ovipositor about 3 times longer than hind tibia; mesopleuron black with two yellow spots separated by a red one.

**Description. Female:** Body length 16.2 mm. Fore wing length 10.8 mm.

**Fig. 1:**
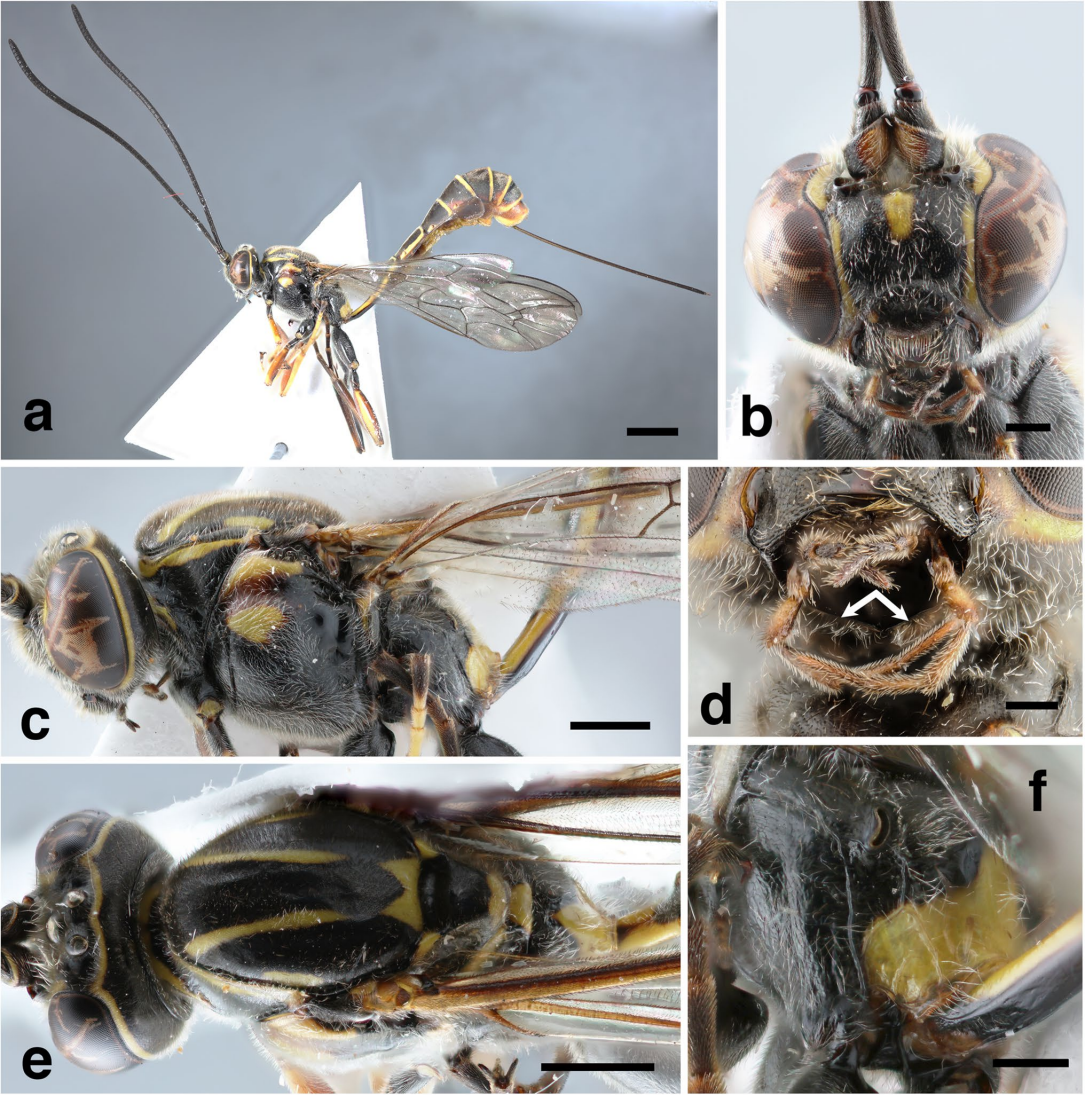
*Grotea akakana* **sp. nov**., female holotype: **a** – habitus; **b** – head (frontal view); **c** – head and mesosoma (lateral view); **d** – head, postgenal process (arrows); **e** – head and mesosoma (dorsal view); **f** – propodeum. Scale bar: 2 mm (**a**); 1 mm (**c, e**); 0.5 mm (**f**); 0.3 mm (**b**); 0.2 mm (**d**)

*Head*. Transverse, 0.5 times as long as wide; gena in dorsal view clearly constricted posterior to eye, slightly rounded, densely and shallowly punctate, shiny with long dense silver setae, about 0.5 times as long as eye (Fig. 1e). Postgenal process present, laterally indistinct (gena with ventroposterior angle modified to form a long spatulate rectangular tooth, both teeth touching each other) (Fig. 1d, arrows). Frons and vertex shiny, densely punctate, slightly convex. Occipital carina complete, rounded dorsally. Posterior ocellus separated from eye by 1.4 times maximum ocellar diameter; distance between posterior ocelli 0.9 times maximum ocellar diameter. Face and clypeus slightly convex, finely and densely punctate on a smooth and shiny background, setae dense, ventral part of clypeus concave, showing labrum (Fig. 1b). Clypeus weakly convex, 2.5 times as wide as long. Ventral tooth of mandible slightly shorter than dorsal tooth. Eye glabrous. Malar space very short, 0.3 times basal mandibular width, about 0.1 times as long as eye in frontal view, smooth and shiny. Antenna with 45 flagellomeres; first flagellomere 5 times as long as wide; flagellum slightly widened towards apex (Fig. 1a).

*Mesosoma*. Entirely covered in dense, long silver setae (Fig. 1c). Pronotum mostly smooth and shiny, very shallowly and densely punctate; epomia distinct. Mesoscutum 1.4 times as long as wide, convex, very densely and shallowly punctate (Fig. 1e); notauli absent; prescutellar groove deep, smooth, without striae; scutellum slightly convex, with same microsculpture as mesoscutum, without lateral carinae. Mesopleuron very densely and shallowly punctate, with very dense and rather long silver setae, setae only absent on speculum; epicnemial carina complete, slightly curved at its dorsal end, ending at level of centre of pronotum; mesopleural suture conspicuous, curved dorsally surrounding yellow spot of mesopleuron. Metapleuron finely punctate, with irregular striae posteriorly, densely pubescent; submetapleural carina strong and complete, forming an obtuse angle anteriorly. Propodeum about 0.7 times as long as broad; anterior transverse carina indented centrally, posterior transverse carina mostly absent, only present between lateral longitudinal and pleural carina, pleural carina complete but thin; propodeum mostly smooth and shiny, setae long and dense (Fig. 1f); area basalis short and wide; area lateralis about 1.1 times as long as broad; spiracle long and arched, touching lateral longitudinal carina. Hind femur and coxa long, length of hind femur 3.9 times its height. Hind tarsal claws thin, evenly curved. Wings with moderately long and dense setae. Fore wing with areolet pentagonal, abscissa of Rs between 2rs-m and 3rs-m 0.3 times as long as vein 3rs-m; vein 2*m-cu* sinuous, with two very short bullae; abscissa of *Cu*1 between 1*m-cu* and *Cu*1*a* about as long as *Cu*1b. Hind wing with distal abscissa of *Cu*1 long, distinctly pigmented to margin of wing; vein *cu-a* + first abscissa of *Cu*1 strongly angled, intercepted clearly above its mid length.

*Metasoma*. Tergite I smooth and shiny, with very few shallow dense setiferous punctures, evenly upcurved, 3.9 times longer than posteriorly broad; thyridia very conspicuous, broad; other tergites with very dense setiferous punctures, more or less shiny. Ovipositor long, about 2.6 times as long as hind tibia; apex of ventral valve of ovipositor covering dorsal valve, with about 7 teeth, without subapical nodus.

*Colour*. Mostly black with yellow marks (Fig. 1a); flagellum black, without a white mark; central spot on face, scapus and pedicel ventrally, a narrow band on orbits (only interrupted at vertex and between face and frons), small lateral spots on clypeus, a narrow continuous band posterior to ocelli confluent with orbits, bands on anterior and dorsal margin of pronotum, two longitudinal bands and two lateral and a posterior mark on mesonotum, posterior part of scutellum, postscutellum, a big anterodorsal spot on mesopleuron (interrupted by a brown band), anterior part of metanotum and a wide posterior band of propodeum, light yellow. Fore and mid legs orange and brown, with coxae and trochanters black; hind legs mostly black with yellow/brown marks on femur and tibia. Metasoma mostly dark brown, tergites I-II with yellow bands surrounding all borders (bands almost touching each other at anterior part of tergite I), and a yellow longitudinal central line from middle of tergite I to anterior third of tergite III; other tergites with yellow band only posteriorly, sometimes a yellow spot anteriorly to spiracle; apex of metasoma somewhat reddish.

**Male**: unknown.

**Etymology** ***.*** The species name refers to Akakana mountain where the holotype was caught; it is part of the cosmovision of Saraguro indigenous people.

**Distribution.** Ecuador.

**Type material.** 1 ♀. **Holotype.** ECUADOR: 1 ♀, Loja, San Lucas, Akakana community, area under restoration, 2940 m, 25-VII/6-VIII/2018, Malaise trap (LOUNAZ).

##### ***Grotea cundinamarquesa*** **Herrera-Flórez, 2018**

**Description of the male:** Body length 12.2 mm. Fore wing 7.2 mm long.

**Fig. 2:**
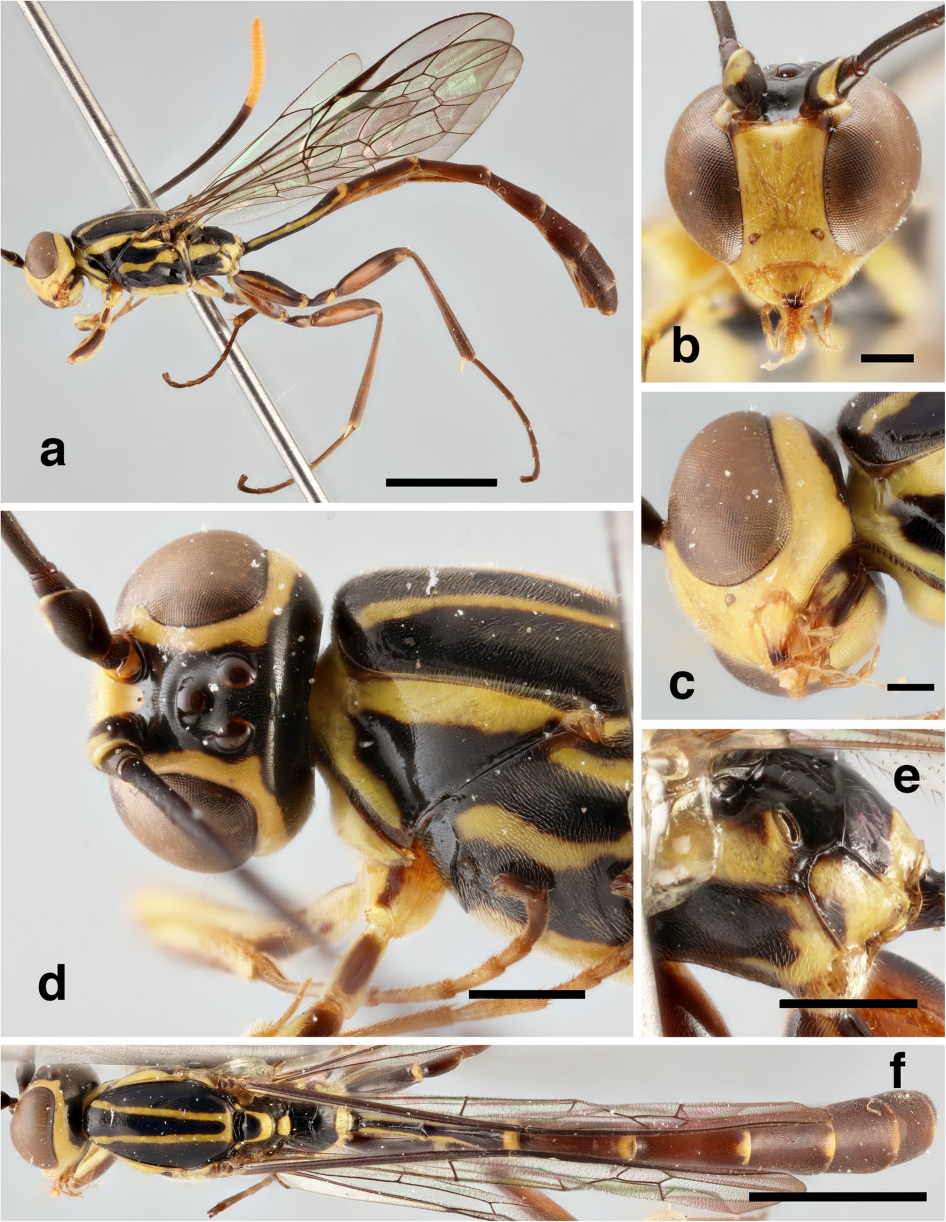
*Grotea cundinamarquesa*, **male nov.**: **a** – habitus (lateral view); **b** – head, frontal view; **c** – head, (lateroventral view); **d** – head (dorsal view) and mesosoma (lateral view); **e** – propodeum (lateral view); **f** – head, mesosoma and metasoma (dorsal view). Scale bar: 2 mm (**a, f**)); 0.5 mm (**d, e**); 0.3 mm (**b**); 0.2 mm (**c**)

*Head*. In dorsal view with gena rounded posterior to eye; posterior ocellus separated from eye by 0.9 times its own maximum diameter (Fig. 2d); postgenal process very short (Fig. 2c). Antenna with 36 flagellomeres.

*Mesosoma*. Mesoscutum smooth with isolated inconspicuous punctures (Fig. 2f); scutellum in profile weakly convex (Fig. 2d); hind wing with *Cu*1 not pigmented to margin; propodeum about 1.0 times as long as broad; anterior transverse carina of propodeum indented centrally, not forming a uniform arc from side to side; pleural carina present posteriorly, replaced anteriorly by a crease (Fig. 2e); posterior transverse carina absent; lateral longitudinal carina anteriorly absent; area spiracularis rectangular; area lateralis rectangular and enclosed, about 1.6 times as long as broad.

*Metasoma*. Tergite I slender, slightly shorter than mesosoma from pronotal collar to posterior margin of propodeum.

*Color*. A black and yellow species (Fig. 2a). Head mostly yellow, frons centrally black, laterally yellow, interocellar area black; gena mostly yellow with hind part black; occiput black; apex of mandibles dark brown; antenna with scape dark brown, ventrally and distally yellow; pedicel and first 26 flagellomeres dark brown, last 10 yellow orange. Mesosoma: pronotum anteriorly and dorsally yellow banded, centrally and posteriorly black; propleuron yellow; mesosternum yellow; mesopleuron mostly black with longitudinal yellow stripes medially and dorsally; mesepimeron yellow; metapleuron mostly black, posteriorly yellow; mesoscutum black, with two longitudinal yellow stripes fused posteriorly; scutellum anteriorly and centrally black, laterally and posteriorly yellow; postscutellum yellow; propodeum with areas basalis, externa, superomedia and dentipara black; areas spiracularis, lateralis, postero-externa, and petiolaris yellow. Fore leg mostly yellow except dorsal longitudinal brown stripes on coxa, trochanter, femur and tibia. Mid leg with anterior side of coxa yellow, dorsal and posterior sides with longitudinal dark brown stripes; trochanter and trochantellus dark brown; femur and tibia dark brown with longitudinal yellow stripe on anterior side; tarsus dark brown. Hind leg with coxa dark brown, ventrally and dorsally with longitudinal yellow stripes; trochanter, femur and tibia dark brown; trochantellus yellow. Wings hyaline. Metasoma with tergite and sternite I black, laterally and posteriorly yellow; tergites II- VIII mostly dark brown, posteriorly yellow medially.

**Distribution.** Colombia, Ecuador*.

**Material examined**. 1 ♂. ECUADOR: 1 ♂, Tungurahua, Baños, 2000 m, 15-IV-1987, M. Cooper., M. Cooper Coll., BMNH(E) 2005-152 1M (NHMUK) (left mid leg glued on label, left antenna broken apically).

##### ***Grotea romeri*Mazón, sp. nov.**

**Diagnosis.** *Grotea romeri* **sp. nov.** can be distinguished from all described species of the genus by the following combination of characters: postgenal process very short; antenna black with 36 flagellomeres, without any white or yellow band; area lateralis of propodeum more than 2 times longer than wide, lateral carina complete; ovipositor about 2.4-2.5 times longer than hind tibia; orbits yellow, not interrupted at vertex; mesopleuron black with a yellow spot in the middle; ovipositor sheath black.

**Description. Female**: Body length 13.5-13.8 mm. Fore wing length 8.7-8.8 mm.

**Fig. 3:**
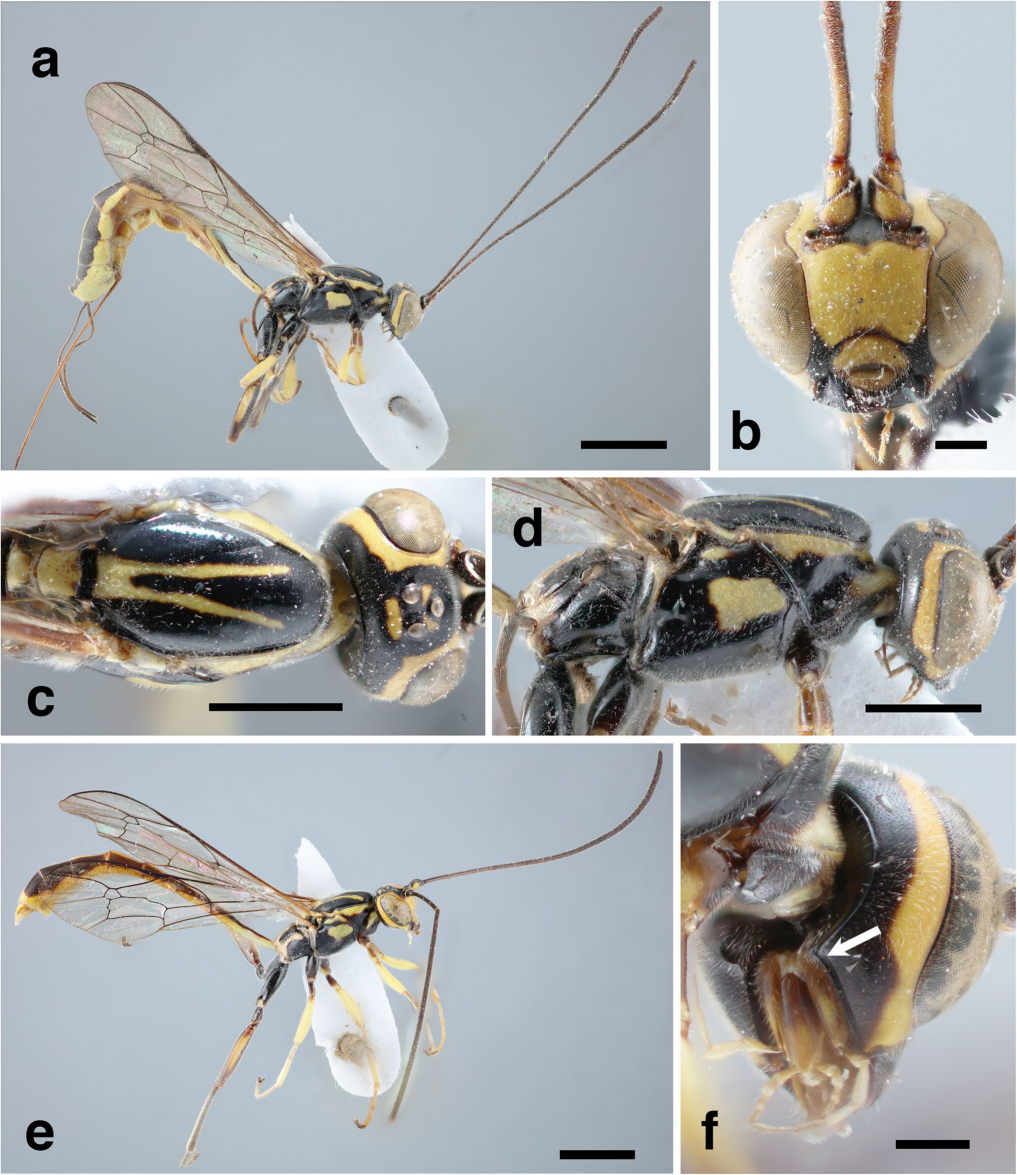
*Grotea romeri* **sp. nov**., female holotype (**a – d**) and male paratype (**e – f**): **a** – habitus (lateral view); **b** – head (frontal view); **c** – head and mesosoma (dorsal view); **d** – head and mesosoma (lateral view); **e** – habitus (lateral view); **f** – head, postgenal process (arrow). Scale bar: 2 mm (**a, e**); 1 mm (**c**, **d**); 0.3 mm (**b**, **f**)

*Head*. Transverse, 0.6 times as long as wide; gena in dorsal view slightly constricted posterior to eye, rounded, smooth and shiny with very short dense setae, about 0.4-0.5 times as long as eye (Fig. 3c). Postgenal process present, laterally indistinct (gena with ventroposterior angle modified to form a short but acute tooth) (Fig. 3f, arrow). Frons and vertex smooth and shiny with very short and dense setiferous punctures, frons conspicuously concave ventrally, dorsally slightly convex. Occipital carina complete, rounded dorsally. Posterior ocellus separated from eye by 1.2-1.7 times maximum ocellar diameter; distance between posterior ocelli 0.7 times maximum ocellar diameter. Face slightly convex, finely, and densely shallowly punctate on a smooth and shiny background, setae very short and dense (Fig. 3b). Clypeus almost flat, 2.5-2.6 times as wide as long, same microsculpture as face, ventral part concave, showing labrum. Mandible teeth subequal in length. Eye glabrous. Malar space 0.4-0.5 times basal mandibular width, about 0.1 times as long as eye in frontal view, same microsculpture as face. Antenna with 36 flagellomeres; first flagellomere 7 times as long as wide; flagellum slightly widened towards apex, filiform.

*Mesosoma*. All mesosoma very shallowly and densely punctate, with very short and dense setae. Pronotum mostly smooth and shiny, very shallowly and sparsely punctate, epomia absent. Mesoscutum 1.7-1.8 times as long as wide, uniformly convex, setae only on perimeter; notauli absent (Fig. 3c); prescutellar groove deep, smooth, without striae; scutellum flat, without lateral carinae. Mesopleuron with speculum smooth and glabrous; epicnemial carina complete, almost straight, reaching anterior margin at ventral third of pronotum; mesopleural suture conspicuous, more or less vertical (Fig. 3d). Metapleuron with same microsculpture as rest of mesosoma; submetapleural carina strong and complete, forming a right angle anteriorly. Propodeum about 1.0 times as long as broad; anterior transverse carina slightly indented centrally, posterior transverse carina absent, pleural carina complete and rather strong; propodeum mostly smooth and shiny, with very fine and shallow setiferous punctures in area lateralis and area spiracularis; area basalis very short and wide; area lateralis very long and narrow, about 3.2-4.0 times as long as broad, almost confluent with area postero-externa, with posterior transverse carina not entirely separating both areas; spiracle long and arched, touching lateral longitudinal and anterior transverse carinae. Hind femur and coxa long, length of hind femur 3.7-4.3 times its height. Hind tarsal claws thin, right-angled curved. Wings with moderately long and dense setae. Fore wing with areolet pentagonal, abscissa of *Rs* between 2*rs-m* and 3*rs-m* 0.6-0.8 times as long as vein 3*rs-m*; 2*m-cu* sinuous, with two very short bullae; abscissa of *Cu*1 between 1*m-cu* and *Cu*1*a* about as long as *Cu*1*b*. Hind wing with distal abscissa of *Cu*1 long, distinctly pigmented until margin of wing; vein *cu-a* + first abscissa of *Cu*1 slightly angled, intercepted at its mid length.

*Metasoma*. Tergite I smooth and shiny, with very dense short setae, very long, 4.2-4.7 times longer than posteriorly broad, clearly upcurved posterior to spiracle; thyridium very short, inconspicuous; other tergites longer than wide, with same microsculpture as tergite I. Ovipositor about 2.0 times as long as hind tibia; apex of ventral valve of ovipositor covering dorsal valve, with about 5 teeth, without subapical nodus.

*Colour*. Mostly black with yellow marks (Fig. 3a); flagellum dark brown, without a white mark; face, clypeus, labrum, scapus, pedicel and first flagellomere ventrally, a wide uninterrupted band on orbits, except ventral part of eyes, triangular marks between anterior and posterior ocelli, a posterior mark posterior to ocelli, bands on anterior and dorsal part of pronotum, two posterior convergent lines on mesoscutum, scutellum, postscutellum, a large central spot on mesopleuron, tegulae, subtegular prominence, mesepisternum, central longitudinal band, posterior margin and anterior spot to spiracle of propodeum, light yellow. Fore and mid legs mostly light yellow, coxae dorsally, trochanters and trochantelli partially dark brown to black; hind legs from dark brown to black, with yellow marks on femur and tibia. Metasoma with all tergites from dark brown to black with median and lateral longitudinal bands.

*Variation*. One of the paratypes has the face centrally black, yellow spots on the interocellar area and vertex less conspicuous, and the median longitudinal line on the first tergite interrupted.

**Male**: Body length 12.3 mm. Fore wing length 7.5 mm.

*Head*. Gena in dorsal view slightly constricted posterior to eye, about 0.7 times as long as eye. Distance between posterior ocelli 0.85 times maximum ocellar diameter. Clypeus 2.2 times as wide as long, ventral margin slightly concave, almost straight. Antenna with first flagellomere 8.2 times as long as wide.

*Mesosoma*. Hind wing with distal abscissa of *Cu*1 not distinctly pigmented to margin of wing.

*Metasoma*. Tergite I 4.0 times longer than posteriorly broad.

*Colour* (Fig. 3e). Mandibles not entirely black, somewhat lighter. Yellow mark on subtegular prominence extended anteriorly. Propodeum with spot on spiracle extended, forming an uninterrupted anteroposterior band.

Other features as in female.

**Etymology.** The species has been named in honour of Oscar Romero, a parataxonomist who has helped to collect and identify most of the material from Ecuador.

**Distribution.** Ecuador.

**Type material.** 2 ♀♀, 1 ♂. **Holotype.** ECUADOR: 1 ♀, Zamora Chinchipe, San Francisco Scientific Station, area under restoration, 1858 m, coordinates 713360 and 9560405, 6-20/I/2016, Malaise trap (LOUNAZ). **Paratypes.** ECUADOR: 1 ♂, Zamora Chinchipe, San Francisco Scientific Station, degraded area, 1839 m, coordinates 713276 and 9560692, 30-XI/17-XII/2015, Malaise trap (LOUNAZ); 1 ♀, Pichincha, Alluligula, 09/05/1992, Leg. V. Yanez (QCAZ) (antennae and ovipositor broken).

#### New records for Ecuador

##### ***Grotea santandereana*** **Herrera-Flórez, 2018**

**Distribution**: Colombia, Ecuador*.

**Material examined**. 3 ♀♀. ECUADOR: 1 ♀, Zamora-Chinchipe, Arcoíris reserve, degraded area, 2161 m, coordinates 711724 and 9558889, 6-20/I/2016, Malaise trap (LOUNAZ); 2 ♀♀, Loja, Madrigal reserve, area under restoration, 2349 m, coordinates 702651 and 9552712, 21-XII-2015/8-I-2016, Malaise trap, (LOUNAZ).

##### ***Grotea surinamese*Herrera-Flórez, 2019**

**Distribution**: Ecuador*, Suriname.

**Material examined**. 2 ♀♀. ECUADOR: 1 ♀, Zamora Chinchipe, El Padmi Biological Station, area under restoration, 822 m, coordinates 764809 and 9586079, 25-II/9-III/2016, Malaise trap (LOUNAZ); 1 ♀, Sucumbíos, Limoncocha, 269 m, 76°37’00”W 00°24’00”S, 11-IV-2005, Leg. D. Céspedes (QCAZ).

#### Key to the Neotropical species of *Grotea*

**Fig. 4:**
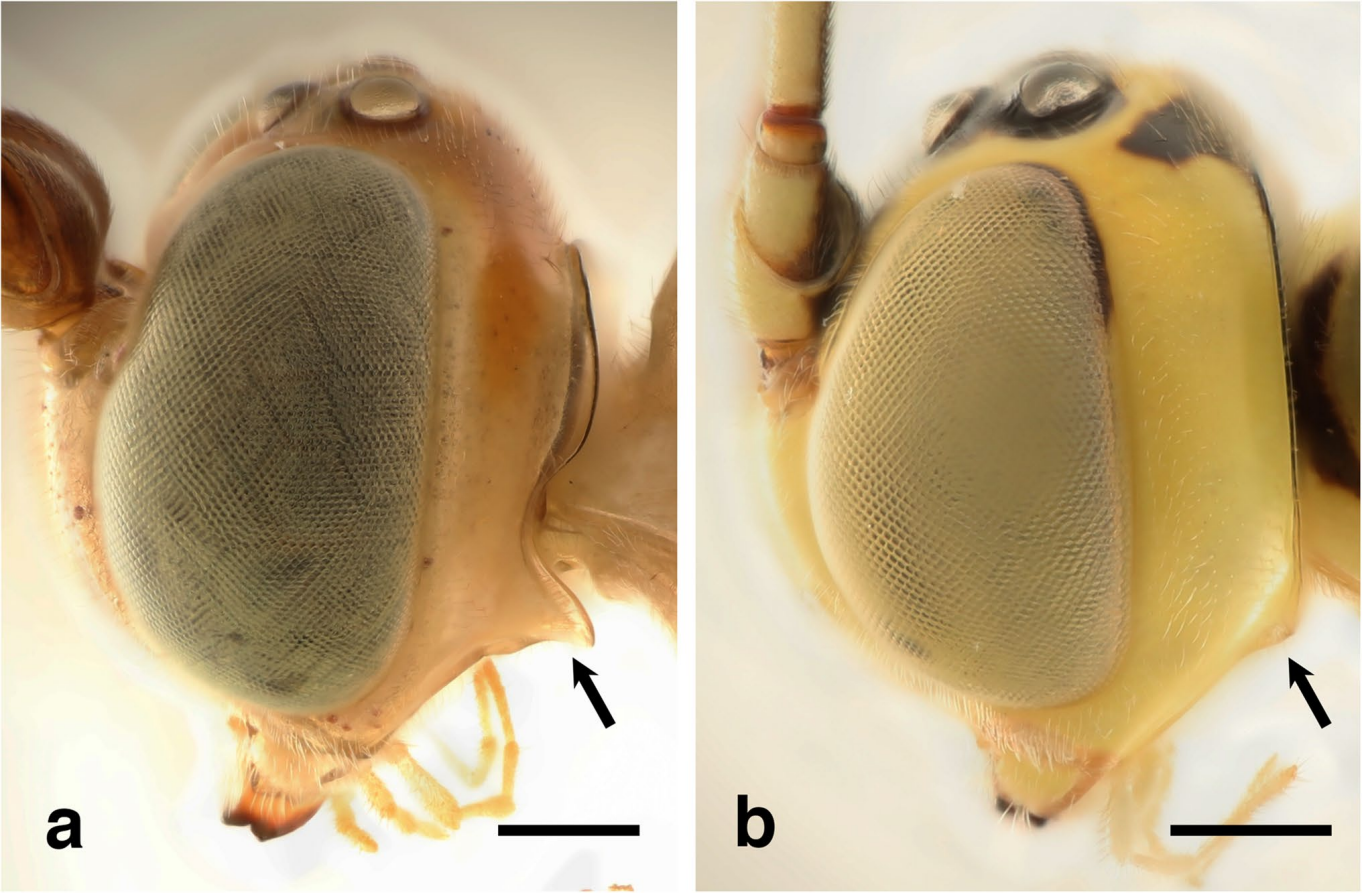
*Grotea* heads showing postgenal process in lateral view: **a** – *G. gracillea* Gauld with postgenal process extended backwards (arrow); **b** – *G. eburnea* Porter with postgenal process horizontally extended, laterally indistinct (arrow). Scale bar: 0.2 mm (**a, b**)

1. Postgenal process extended downwards or backwards, laterally distinct (Fig. 4a, arrow)2

**Fig. 5:**
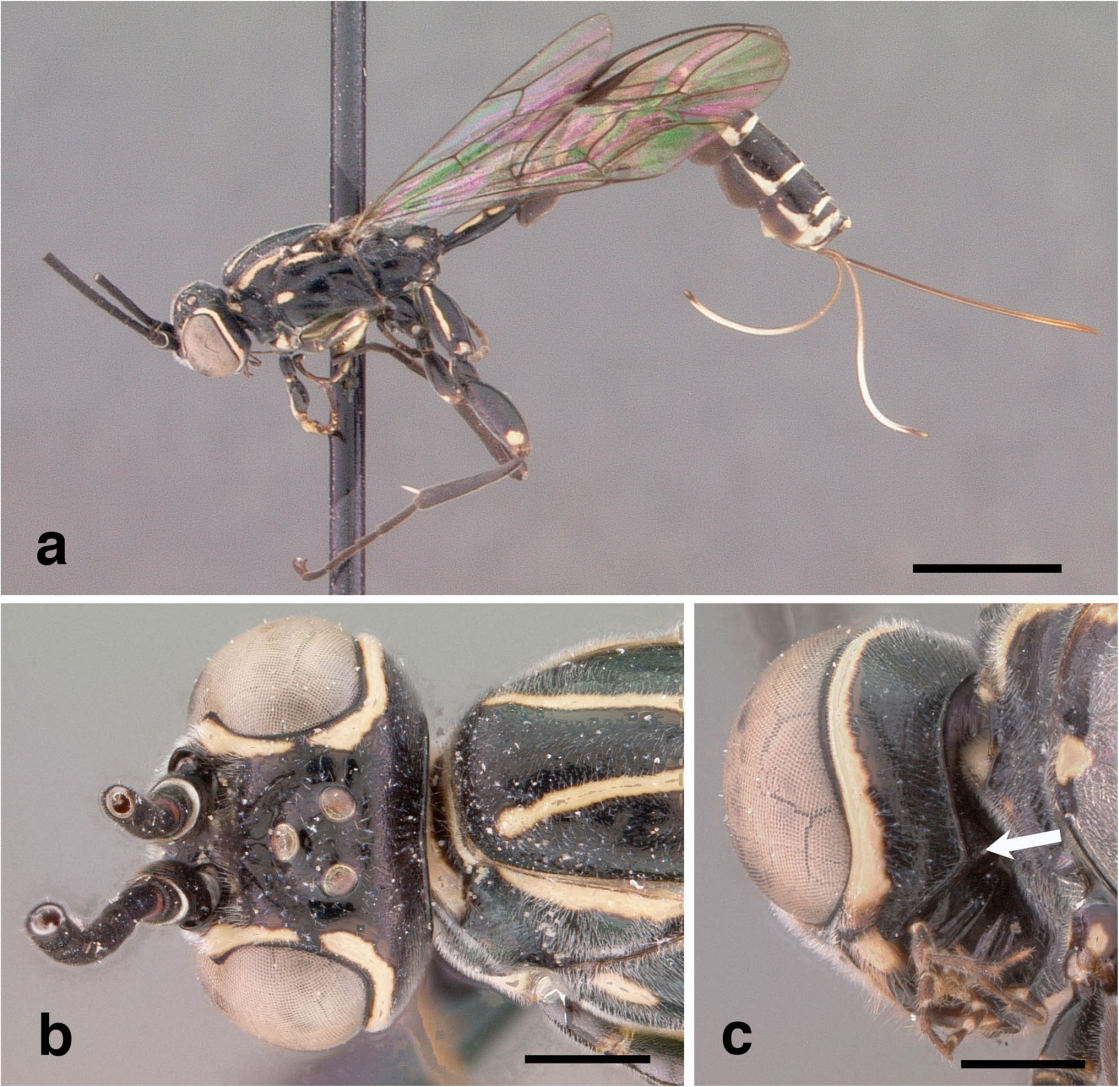
*Grotea cortesi* Porter, female holotype: **a** – habitus (lateral view); **b** – head and mesosoma (dorsal view); **c** – head, postgenal process (arrow). Scale bar: 2 mm (**a**); 0.5 mm (**b**, **c**)

-. Postgenal process horizontally extended, laterally indistinct (Figs. 1d, 3f, 4b, 5c, arrows)14

2. Mesopleuron with black and white stripes, or mostly black with yellow marks. Flagellum with white band. Postgenal process with acute apex3

-. Mesopleuron entirely yellow, orange or red, or mostly yellow with longitudinal orange or black stripes, or with red or black spots anteriorly and posteriorly. Flagellum with or without white band. Postgenal process various4

3. Gena short, slightly concave in dorsal view. Mesopleuron black in the middle, with anterior and posterior yellow spots. Fore wing with distal dark spot. Flagellum dark brown to black with white band on flagellomeres 32-40

***G. surinamese*** **Herrera-Flórez, 2019**

-. Gena slightly convex in dorsal view. Mesopleuron with longitudinal black and white stripes. Fore wing entirely hyaline. Flagellum dorsally black, white ventrally, with a white band on flagellomeres 24-42 ***G. superba*** **Schmiedeknecht, 1907**

4. Mesopleuron entirely red. Flagellum dark brown or black without white band5

-. Mesopleuron entirely yellow, or mostly yellow with orange, black or red marks. Flagellum with or without white band6

5. Gena convex in dorsal view. Antenna with 40 flagellomeres. Anterior transverse carina of propodeum not indented, gently arched

***G. ambarosa*** **Sandoval and Santos,** **2021**

-. Gena straight in dorsal view. Antenna with 49 flagellomeres. Anterior transverse carina of propodeum indented in the center ***G. paulista*** **Herrera-Flórez, 2019**

6. With the following combination of characters: antenna with 46-47 flagellomeres, flagellum yellowish brown to dark brown, distally grading to black, with a short subdistal white band; gena narrowed posterior to compound eye, not convex in dorsal view; postgenal process elongate, rounded apically (Fig. 4a, arrow); mesopleuron yellow with a posterior black mark ***G. gracillea*** **Gauld, 2000**

-. Not all those characters combined7

7. Gena convex in dorsal view. Flagellum yellow-brown with a subapical black band and white apex ***G. athenae*** **Slobodchikoff, 1970**

-. Gena straight or slightly concave in dorsal view. Flagellum without a white band, sometimes brown-yellow with black apex 8

8. Fore wing with distal black spot. Mesopleuron yellow with 1-2 black marks. Antenna very long, with 52-56 flagellomeres9

-. Fore wing entirely hyaline, without distal black spot. Mesopleuron entirely yellow, or yellow with red marks (sometimes yellow with black spots anteriorly and posteriorly in *G. claudiae*). Antenna not as long, with fewer than 50 flagellomeres (except for *G. fulva*, and unknown for *G. claudiae*)10

9. Pleural carina weak or absent from the transverse carina to the posterior rim of propodeum. Postgenal process wide at the base, tapering towards apex. Antenna dark brown to black ***G. carara*** **Gauld, 2000**

-. Pleural carina strong along its entire length. Postgenal process subrectangular. Antenna brown-yellow with black apex ***G. vanessae*** **Gauld, 2000**

10. Ovipositor very long, 2.7-2.9 times length of hind tibia. Area lateralis of propodeum more than 3 times as long as wide. Gena straight in dorsal view. Antenna brown-yellow with black apex ***G. djinnae*** **Gauld, 2000**

-. Ovipositor less than 2.2 times length of hind tibia. Area lateralis of propodeum less than 2.5 times as long as wide. Gena from straight to slightly concave in dorsal view. Antenna from yellow to dark brown or black, without a black apex

11

11. Ovipositor very short, about 1.3-1.4 times length of hind tibia. Mesopleuron entirely yellow. Postgenal process subrectangular ***G. delicator*** **Thunberg, 1822**

-. Ovipositor longer, at least 2 times length of hind tibia. Mesopleuron yellow with or without black marks or red and yellow. Postgenal process tapered towards the apex

12

12. Antenna yellow or light brown. Gena straight in dorsal view, occipital carina not raised in the middle, pronotum not particularly exposed. Posterior corner of area lateralis very acute ***G. fulva*** **Cameron, 1886**

-. Antenna dark brown to black (sometimes proximally lighter). Gena in dorsal view slightly concave, with occipital carina slightly rised into a lamella in the middle and pronotum very exposed. Posterior corner of area lateralis right angled13

13. Mesopleuron red and yellow. Fore wing with abscissa of *Cu*1 between 1*m-cu* and *Cu*1*a* forming a distinct obtuse angle with *Cu*1*b*.

***G. goianiense*** **Herrera-Flórez, 2019**

-. Mesopleuron yellow with black marks. Fore wing with abscissa of *Cu*1 between 1*m-cu* and *Cu*1*a* + *Cu*1*b* straight or forming an even arch ***G. claudiae*** **Kumagai, 2024**

14. Mesopleuron red with yellow or black marks. Antenna dark brown to black, without white band15

-. Mesopleuron mostly yellow with red or black marks, or white with black marks, or black with yellow marks. Antenna with or without white or yellow marks

17

15. Mesopleuron red with two large black spots. Ovipositor long, about 2.7-2.9 times length of hind tibia. Gena rounded in dorsal view

***G. villosissima*** **Herrera-Flórez, 2014**

-. Mesopleuron red with yellow marks. Ovipositor shorter, less than 2.4 times length of hind tibia. Gena straight or slightly concave in dorsal view16

16. Mesopleuron red with a yellow spot on antero-dorsal part. Ovipositor about 2 times as long as hind tibia. Anterior transverse carina of propodeum gently arched, not indented medially. Gena slightly concave in dorsal view

***G. perplexa*** **Slobodchikoff, 1970**

-. Mesopleuron red with a longitudinal central yellow band. Ovipositor about 2.4 times length of hind tibia. Anterior transverse carina of propodeum indented medially. Gena straight in dorsal view ***G. santandereana*** **Herrera-Flórez, 2018**

17. Mesopleuron mostly yellow with black marks18

-. Mesopleuron black and white, or mostly black with yellow marks (Figs. 1c, 2a, 3d, 5a)

20

18. Malar space long, about 0.9 times basal mandibular width. Antenna with 31 flagellomeres, without white band ***G. fernandoi*** **Lima, 2024**

-. Malar space shorter, less than 0.5 times basal mandibular width. Antenna with more than 31 flagellomeres, with white band19

19. Mesopleuron yellow with two longitudinal black bands. Gena convex in dorsal view. Antenna with 40 flagellomeres, white band on flagellomeres 22-31. Area lateralis of propodeum rectangular, about 3 times as long as wide. Pleural carina of propodeum absent. Ovipositor about 1.7 times length of hind tibia.

***G. llanera*** **Herrera-Flórez, 2018**

-. Mesopleuron yellow with black spots on anterior and posterior parts. Gena slightly concave in dorsal view. Antenna with 51 flagellomeres, white band on flagellomeres 32-40. Area lateralis about 1.5 times as long as wide. Pleural carina of propodeum present. Ovipositor 2.2-2.5 times length of hind tibia.

***G. manausi*** **Herrera-Flórez, 2019**

20. Postgenal processes long, almost touching (Fig. 1d, arrow) 21

-. Postgenal process very short (Figs. 3f, 5c arrows)24

21. Mesopleuron black with two yellow spots separated by a reddish one (Fig. 1c). Ovipositor very long, about 3 times length of hind tibia (Fig. 1a). Antenna long, about 45 flagellomeres, without white band (Fig. 1a) ***G. akakana*** **sp. nov.**

-. Mesopleuron black and white. Ovipositor shorter. Antenna shorter, fewer than 38 flagellomeres, with white band22

Postgenal process acute. Antenna black on dorsal part, white on ventral part, white band on flagellomeres 22-28. Mesopleuron white with a longitudinal black band, usually interrupted in the middle.***G. eburnea*** **Porter, 1989**

-. Postgenal process subrectangular. Antenna entirely dark brown or black, white band longer or starting before. Mesopleuron with different colour pattern23

Lateral longitudinal carina of propodeum weak or absent posteriorly to spiracle. White band on antenna long, present on flagellomeres 18(20)-34(35). Mesopleuron mostly black with white marks.***G. chiloe*** **Porter, 1989**

-. Lateral longitudinal carina of propodeum complete. White band on antenna shorter, present on flagellomeres 24-30(32). Mesopleuron mostly white with black marks.

***G. oneilli*** **Porter, 1989**

Mesopleuron white with longitudinal black bands. Ovipositor very long, about 5.4 times length of hind tibia, ovipositor sheath white. Lateral longitudinal carina of propodeum only present posterior to transverse carina, very weak. Antenna with about 41 flagellomeres and white band on flagellomeres 25-34

***G. gayi*** **Spinola, 1851**

-. Mesopleuron black with yellow marks. Ovipositor shorter, less than 2.5 times length of hind tibia, ovipositor sheath from white to dark brown to black. Lateral longitudinal carina of propodeum complete. Antenna without white band, or with distal 16-17 flagellomeres yellow25

25. Ovipositor sheath white (Fig. 5a). Mesopleuron mostly black, with only a couple of small yellow spots (Fig. 5a). Eye orbits narrowly yellow, briefly interrupted at vertex (Fig. 5b, c). Ovipositor about twice length of hind tibia (Fig. 5a).

***G. cortesi*** **Porter, 1989**

-. Ovipositor sheath brown or black. Mesopleuron with one or more yellow marks covering more than 25% of the surface (Figs. 2a, 3d, e). Eye orbits widely yellow, not interrupted at vertex. Ovipositor about 2.4-2.5 times length of hind tibia26

26. Antenna black with 39 flagellomeres, distal 16-17 flagellomeres yellow. Mesopleuron black with longitudinal yellow stripes (Fig. 2a). Area lateralis of propodeum about 1.3 times as long as wide

***G. cundinamarquesa*** **Herrera-Flórez, 2018**

-. Antenna black with 36 flagellomeres, without any white or yellow band (Fig. 3a, e). Mesopleuron black with a yellow spot in the middle (Fig. 3d, e). Area lateralis of propodeum more than 2 times as long as wide ***G. romeri*** **sp. nov.**
